# Evaluation of risk for bronchiolitis obliterans syndrome after allogeneic hematopoietic cell transplantation with myeloablative conditioning regimens

**DOI:** 10.1038/s41409-024-02422-z

**Published:** 2024-09-27

**Authors:** Jesús Duque-Afonso, Paraschiva Rassner, Kristin Walther, Gabriele Ihorst, Claudia Wehr, Reinhard Marks, Ralph Wäsch, Hartmut Bertz, Thomas Köhler, Björn Christian Frye, Daiana Stolz, Robert Zeiser, Jürgen Finke, Kristina Maas-Bauer

**Affiliations:** 1 Department of Hematology/Oncology/Stem Cell Transplantation, Faculty of Medicine and Medical Center—University of Freiburg, Freiburg, Germany; 2 Clinical Trials Unit, Faculty of Medicine and Medical Center—University of Freiburg, Freiburg, Germany; 3 Clinic of Respiratory Medicine, Faculty of Medicine and Medical Center—University of Freiburg, Freiburg, Germany

**Keywords:** Translational research, Risk factors

## Abstract

Bronchiolitis obliterans syndrome (BOS), as chronic manifestation of graft-versus-host disease (GVHD), is a debilitating complication leading to lung function deterioration in patients after allogeneic hematopoietic cell transplantation (allo-HCT). In the present study, we evaluated BOS development risk in patients after receiving myeloablative conditioning (MAC) regimens. We performed a retrospective analysis of patients undergoing allo-HCT, who received MAC with busulfan/cyclophosphamid (BuCy, *n* = 175) busulfan/fludarabin (FluBu4, *n* = 29) or thiotepa/busulfan/fludarabine (TBF MAC, *n* = 37). The prevalence of lung disease prior allo-HCT, smoking status, GvHD prophylaxis, HCT-CI score, EBMT risk score and GvHD incidence varied across the groups. The cumulative incidence of BOS using the NIH diagnosis consensus criteria at 2 years after allo-HCT was 8% in FluBu4, 23% in BuCy and 19% in TBF MAC (*p* = 0.07). In the multivariate analysis, we identified associated factors for time to BOS such as FEV1<median (99% of predicted) (HR = 2.39, *p* = 0.004), CMV patient serology positivity (HR = 2.11, *p* = 0.014), TLC < 80% of predicted (HR = 0.12, *p* = 0.02) and GvHD prophylaxis with in vivo T-cell depletion (HR = 0.29, *p* = 0.001) as predictors of BOS. In summary, we identified risk factors for BOS development in patients receiving MAC conditioning. These findings might serve to identify patients at risk, who might benefit from closely monitoring or early therapeutic interventions.

## Introduction

Chronic graft versus host disease (cGvHD) remains one of the leading causes for morbidity and mortality following allogeneic hematopoietic cell transplantation (allo-HCT). Although recent advances elucidating the basic, preclinical, and clinical biology of chronic GvHD have been made, effective preventive and treatment options are limited [1, 2]. Furthermore, among the multiple organs involved in cGvHD, pulmonary cGvHD is especially difficult to treat [3].

Although late interstitial pneumonitis (IP) and cryptogenic organizing pneumonia (COP) are often associated with cGvHD [4–6], BOS is the only manifestation considered diagnostic of cGvHD [7, 8] and results from the immune reaction in the small terminal airways, leading to fibrotic remodeling and occlusion [9]. Therapy is directed to stabilize the disease, for which currently most experts prefer regimens consisting of systemic or inhaled corticosteroids, long-acting β-2 agonists, azithromycin, and leukotriene receptor antagonists [10–13]. To harmonize the definition of BOS for comparative studies and clinical trials, the National Institutes of Health (NIH) has defined and developed consensus diagnostic criteria for BOS after allo-HCT [7, 14]. Furthermore, pretransplant obstructive lung disease have been linked to worse outcome in patients undergoing allo-HCT [15, 16] and pretransplant restrictive lung disease has been associated with early respiratory failure [17] and long-term complications [18] in the context of allo-HCT.

A combination of busulfan and cyclophosphamide (BuCy) was the first prototype of chemotherapeutic myeloablative conditioning (MAC) [19]. Later conditioning with fludarabin and busulfan (FluBu4) was introduced [20]. To further enhance the antileukemic effects of these protocols, several chemotherapeutic combinations were established. Among them, the combination of thiotepa, busulfan and fludarabin (TBF MAC) has been widely used [21–24]. In previous studies, we described and characterized clinical risk factors for BOS and investigated the impact of BOS on the outcome of patients undergoing allo-HCT with reduced toxicity/intensity conditioning (RIC) [25, 26]. In the present study, we evaluated the risk of BOS across patients conditioned with myeloablative regimens (BuCy, FluBu4, TBF MAC).

## Methods

### Study design

In this retrospective analysis, patients with acute myeloid leukemia (AML), myelodysplastic syndrome (MDS) and myeloproliferative syndromes (MPN) undergoing first allo-HCT at the University of Freiburg Medical Center were included. The inclusion criteria were: (1) adult (aged > 18 years) patients who received MAC with Busulfan/Cyclophosphamid (BuCy, Busulfan i.v. 3.2 mg/kg/day from day -7 to day -4, cyclophosphamide 60 mg/kg/day i.v. from day -3 to -2), Busulfan/ Fludarabin (FluBu4, Fludarabin 30 mg/m^2^/day from day -7 to day -4, Busulfan 3,2 mg/kg/day from day -7 to day -4) or TBF MAC (Thiotepa 5 mg/kg/day from day -7 to day -6, Fludarabin 30 mg/m^2^ d-5 to d-3, Busulfan 3,2 mg/kg/day from day -5 to day -3). (2) All patient with a first allo-HCT were included in this study, (3) allo-HCT from a matched sibling donor (MSD) or matched or mismatched unrelated donor (MUD 10/10 and MMUD 9/10—including HLA-A, -B, -C, or -DRB1 and DQB1 mismatches -) (4) transplantation date between January 1st, 1998 and September 30th, 2019, (5) with an unmanipulated peripheral blood stem cell graft (no in vitro T-cell depletion (TCD)). Patients undergoing haplo-identical or cord blood allo-HCT and patients with a syngeneic donor were also excluded. CR was defined as less than 5% blasts in bone marrow at the time of allo-HCT. Cyclosporine-based GvHD prophylaxis (cyclosporine 5 mg/kg body weight per day, starting day 3, targeted through serum level) was combined with methotrexate (15 mg/m^2^ on day +1, 10 mg/m^2^ on days +3 and +6), mycophenolate mofetil (2 × 1 g), antithymocyte globulin (ATLG-Grafalon (earlier Fresenius); 30 to 60 mg/kg body weight) [27, 28], prednisolone or alemtuzumab (given i.v., at day -2 and -1, ranging from 10 to 40 mg) [29]. Patients receiving BuCy underwent transplantation between January 1, 1998, and April 30, 2012, patients receiving FluBu4 or TBF MAC underwent transplantation between January 1, 2014 and September 30, 2019. Conditioning protocols FluBu4 and TBF MAC were selected by the caring physicians according to patient and disease characteristic including remission status prior allo-HCT, genetic markers and were not randomized. Post-transplant events such as hematological relapse and GvHD were defined based on standard clinical and laboratory criteria. All clinical data were prospectively collected and retrospectively analyzed. The investigators were not blinded during the outcome analysis. All data were evaluated as of December 31th 2023.

### Ethics approval and consent to participate

This study was conducted in accordance with the amended Declaration of Helsinki. The institutional review board of the University of Freiburg Medical Center approved this study (study protocol Nr. 22-1490-S1-retro) and written informed consent was obtained from the subjects for data use in clinical research.

### Pulmonary function tests

Patients were clinically examined weekly or every 2 weeks during the first 3 months after transplantation. PFTs were routinely performed a week before allo-HCT and after 3, 6, 12, and 24 months, then repeated on the basis of clinical suspicion of pulmonary disease. PFT parameters were evaluated and expressed as percentage of predicted normal values, calculated using published equations [30]. The diagnosis of BOS was defined by NIH criteria [7, 14, 31]. To exclude infection, thorax CT and standard culture and staining methods for bacterial, viral, and fungal pathogens were used routinely from body fluids including sputum and bronchoalveolar lavages. Each PFT from each patient was verified individually.

### Statistical analysis

Outcome variables such as overall survival (OS), progression-free survival (PFS), relapse and non-relapse mortality (NRM) were defined following internal consensus guidelines [32]. Patient-, disease- and treatment-related characteristics were compared using the chi-square test for categorical data or analysis of variance (ANOVA) and Student’s t-test for continuous data. Paired comparisons were conducted with Wilcoxon’s matched-pair signed-rank test for continuous variables. Baseline characteristics were summarized using median and range for continuous data, and frequency and percentage for categorical data. OS was defined as the time from allo-HCT until death from any cause. PFS was defined as the time from allo-HCT to death from any cause, or relapse, whichever occurred first. Relapse was defined as detection of disease via cytological and histological assessment after allo-HCT; death without prior relapse was considered as a competing risk for relapse and was denoted as NRM. For cumulative incidence of BOS, acute GvHD (aGvHD) and cGvHD, death without BOS/aGvHD/cGvHD, respectively, were considered as competing events. Patients with no events were censored at the date of last follow-up. The median follow-up was calculated using the inverse Kaplan Meier method [33]. Univariate analyses were performed using Gray’s test for cumulative incidence functions as relapse, NRM, BOS, aGvHD and cGvHD [34, 35] and the log-rank test for OS and PFS. The Cox proportional-hazards model and Fine and Gray regression model for competing risks were used for multivariate regression analysis backward selection process of prognostic factors with a univariate *p* value < 0.1. We included the type of myeloablative conditioning (BuCy, FluBu4, TBF MAC) as hypothesis confounding variable in multivariate analysis. Results were expressed as the hazard ratios (HRs) and subdistribution hazard ratios (SHR) with 95% confidence intervals (95% CI). All tests were two sided. The Type I error was fixed at 0.1 for factors associated with time-to-event outcomes. Statistics were performed with STATA v17.0 (College Station, Texas, USA).

## Results

### Clinical features of patients treated with myeloablative conditioning prior allo-HCT

The clinical and transplant characteristics of the 241 patients included in this study are shown in Table 1 and Supplementary Table 1. Knowing that conditioning prior allo-HCT has a significant impact on pulmonary function and complications after allo-HCT including BOS development, we focused first on the analysis of patient characteristics of each conditioning cohort. Prior to allo-HCT, 175 patients received a conditioning with BuCy, 29 patients with FluBu4 and 37 patients with TBF MAC. The median patient age was 43 years in the BuCy, 46 years in the FluBu4 and 39 years in the TBF MAC group and the median follow-up was 65, 62 and 40.3 months, respectively. In terms of comorbidities and disease risk score, we observed a lower HCT-CI score but a higher EBMT disease risk score in the BuCy cohort, compared to the two other groups (Supplementary Table 1). Regarding GvHD-prophylaxis based on in vivo TCD, there were also significant imbalances between the three conditioning groups: alemtuzumab was only used in the BuCy (22%) cohort and ATG was more often used in FluBu4 and TBF MAC conditioning (41% vs. 59% vs 78%, respectively) (Table 1).

**Table 1:** Clinical characteristics and pulmonary function tests at the time of allo-HCT.

	BuCy	FluBu4	TBF MAC	*p* value
*N*	175	29	37	
Patient sex (% of male)	99 (57)	16 (55)	19 (51)	0.84
Donor sex (% of male)	101 (58)	20 (69)	18 (49)	0.25
Age at allo-HCT, median (range)	43 (18, 58)	46 (26, 59)	39 (19, 59)	0.08
KPS, median (range)	90 (30, 100)	90 (30, 100)	90 (70, 100)	0.77
Median follow up in months (range)	65 (3, 275)	62 (2, 107)	40.3 (2, 100)	0.001
**Donors, n (%)**				
- related	61 (35)	9 (31)	10 (27)	0.63
- unrelated	114 (65)	20 (69)	27 (73)	
**GvHD prophylaxis, n (%)**				
- without in vivo TCD	65 (37)	12 (41)	8 (22)	8 (22)
- with in vivo TCD	110 (63)	17 (59)	29 (78)	0.15
**Type of GvHD prophylaxis**, n (%)				
- CyA/ATG	72 (41)	17 (59)	29 (78)	0.001
- CyA/alemtuzumab	38 (22)	0	0	
**Lung disease before allo-HCT, n (%)**	74 (42)	1 (4)	7 (19)	0.001
- fungal pneumonia or aspergillosis	31	1	1	
- bact. pneumonia	15	0	2	
- bronchitis or upper airway infection	11	0	0	
- COPD/Asthma	8	0	0	
- atypical pneumonia	3	0	1	
- viral pneumonia	2	0	1	
- pleura effusions	4	0	0	
- pulmonary embolism	4	0	0	
- others (leukemia infiltration, pleuritis, aspiration pneumonia, interstitielle pneumonia, lung edema, restrictive lung disease)	4	0	2	
**Lung disease up to 100 d after allo-HCT, n (%)**	49 (28)	5 (17)	7 (19)	0.29
- bact. pneumonia	21	0	2	
- fungal pneumonia or aspergillosis	12	2	3	
- obstructive lung function	7	0	0	
- viral pneumonia	4	1	1	
- bronchitis or upper airway infection	4	1	0	
- pleura effusions	5	0	1	
- others (engraftment syndrome, atypical pneumonia)	8	1	0	
**Smoking current or previous (%)**	53 (30)	3 (10)	2 (5)	0.001
**Pulmonary function tests before allo-HCT, median (range)**				
- FEV1 (% predicted)	98 (44, 142)	99 (70, 133)	98 (67, 127)	0.88
- FEV1/FVC ratio	0.83 (0.55, 1.00)	0.82 (0.66, 0.95)	0.84 (0.67, 1.13)	0.75
- MEF50 (% predicted)	70 (4, 159)	81 (5, 130)	82 (44, 147)	0.07
- MEF25 (% of predicted)	45 (11, 122)	57 (2, 124)	53 (18, 242)	0.003
- DLCOc SB (% predicted)	76 (46, 109)	74 (52, 108)	76 (40, 1.04)	0.63
- RV (% predicted)	111 (42, 223)	107 (64, 153)	103 (55, 148)	0.18
- RV/TLC ratio	0.33 (0.14, 1.18)	0.34 (0.19, 0.49)	0.30 (16, 108)	0.18
- TLC (% predicted)	96 (51, 131)	96 (79, 130)	97 (65, 133)	0.17
- aCO2 (mmHg)	37 (27, 46)	36 (30, 44)	37 (28, 42)	0.15
- aO2 (mmHg)	83 (65, 103)	84 (67, 92)	85 (66, 106)	0.005
**Time from allo-HCT to BOS diagnosis in months, median (range)**	12.5 (2.4, 111.3)	8.91 (3.6, 14.2)	13.6 (5.5, 30.4)	0.75
**Cumulative incidence of BOS after allo-HCT, % (95% CI) at**				**0.07**
- 1 year	11.9 (7.7, 18.3)	3.6 (0.5, 22.7)	9.3 (3.1, 26.1)	
- 2 year	23.2 (17.0, 31.1)	8.4 (2.1, 30.1)	19.5 (9.2, 38.6)	
- 3 year	30.2 (23.1, 38.9)	8.4 (2.1, 30.1)	23.4 (11.0, 43.1)	
- 5 year	31.2 (23.9, 39.9)	8.4 (2.1, 20.1)	23.4 (11.0, 43.1)	
**BOS severity, n (%)**				
- Mild (FEV1 60-79%)	12 (26)	–	(28)	
- Moderate (FEV1 40-59%)	21 (47)	2 (100)	(57)	
- Severe (FEV1 ≤ 39%)	12 (26)	–	1 (14)	

*Allo-HCT* allogeneic hematopoietic cell transplantation, *KPS* Karnofsky performance score, *GvHD* graft-versus-host disease, *TCD* T-cell depletion, *CyA* cyclosporine A, *ATG* antithymocyte globuline, *COPD* chronic obstructive pulmonary disease, *bact.* bacterial, *BOS* bronchiolitis obliterans syndrome, *FEV1* forced expiratory volume in 1 s (FEV1), *FVC* forced vital capacity, *RV* residual volume, *TLC* total lung capacity, *MEF50* mid-expiratory flow 50%, *MEF25* mid-expiratory flow 25%, *DLCOc SB* carbon monoxide diffusion capacity corrected for hemoglobin, *BuCy* Busulfan/Cyclophosphamide, *FluBu4* Fludarabine/Busulfan 4 days, *TBF MAC* Thiotepa/Busulfan/Fludarabine myeloablative conditioning.

### Pulmonary characteristics and complications prior and after allo-HCT

There were significant differences in the pulmonary clinical features across the different conditioning groups (Table 1): (1) Patients in the BuCy group had the highest prevalence of lung diseases prior to allo-HCT (42% vs. 4% and 19%, respectively, *p* < 0.001) compared to patients in the other two groups. (2) The rate of current or previous smokers was the highest in the BuCy group (30% vs 10% and 5%, respectively, *p* = 0.001) compared to patients in the FluBu4 and TBF MAC group. (3) Pulmonary evaluation prior to allo-HCT differed numerically in regard to two PFT parameters: MEF25 (45% vs 57% vs 53%, *p* = 0.003) and arterial O_2_ (83 vs 84 vs 85 mmHg, *p* = 0.005) between groups.

### BOS diagnosis and impact on outcome after allo-HCT

We analyzed the cumulative incidence of BOS using the NIH diagnosis consensus criteria [7, 14, 31] in patients conditioned with MAC regimens (Table 1, Supplementary Fig. 1). Strikingly, we observed a trend for lower BOS incidence in patients receiving FluBu4 compared to patients that received either BuCy or TBF MAC as conditioning regimen (Table 1): At 1 year, BOS incidence was 3.6% in patients treated with FluBu4, 11.9% in BuCy and 9.3% in TBF MAC and at 5 years, it was 8.4% in FluBu4 cohort compared to 31.2% in the BuCy and 23.4% in the TBF MAC cohort. Six patients developed BOS prior d + 100 and 5 patients developed cGvHD prior d + 100. Moreover, using the NIH criteria we found that most of the patients had already moderate severity of BOS at diagnosis: in the BuCy group 12 patients (26%) had severe and 21 patients (47%) moderate BOS; in the TBF MAC group 1 patient (14%) had a severe degree and 4 patients (57%) a moderate degree of BOS. In the FluBu4 group, we observed only 2 that were diagnosed with moderate severity of BOS. Pulmonary function parameters were analyzed prior allo-HCT and at BOS diagnosis only in patients, who had a suspicion of BOS (Supplementary Table 2). Almost all analyzed parameters except TLC deteriorated between pre-transplant assessment and assessment at BOS diagnosis.

**Fig. 1:**
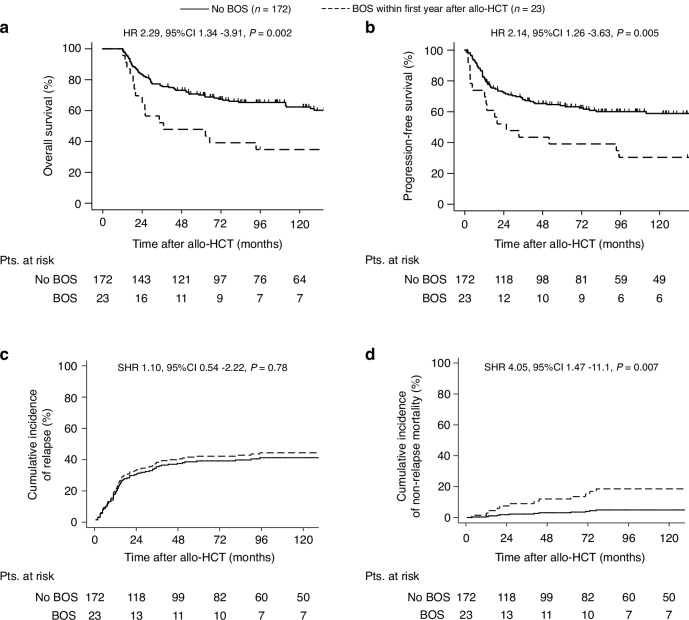
Outcome variables by BOS development within the first year after allo-HCT. To address the influence of BOS development on outcome after allo-HCT we performed a landmark analysis. Patients surviving 365 days after allo-HCT were included in the analysis (*n* = 195). Outcome variables were analyzed by BOS development within the first year after allo-HCT. Kaplan-Meier curves represent (**a**) overall survival and (**b**) progression-free survival and cumulative incidence curve represent (**c**) non-relapse mortality and (**d**) relapse incidence in patients conditioned with myeloablative conditioning regimens prior allo-HCT. Pts patients, allo-HCT allogeneic hematopoietic cell transplantation.

To estimate the impact of BOS on outcome variables of patients after allo-HCT, we performed a landmark analysis for patients surviving at least 1 year after allo-HCT (*n* = 195). We analyzed outcome variables by BOS development within the first year after allo-HCT (Fig. 1). Patients developing BOS within the first year after allo-HCT had a decreased OS (HR 2.29, 95% CI 1.34–3.91, *p* = 0.002) and PFS (HR 2.14, 95% CI 1.26–3.63, *p* = 0.005) and increased NRM (SHR 4.05, 95% CI 1.47–11.1, *p* = 0.007). As expected, we found no differences on relapse incidence between both groups (SHR 1.1, 95% CI 0.54–2.22, *p* = 0.78). Of note, we could not assess the impact of BOS severity on outcome due to the low number of patients in the subgroup analysis (*n* = 6 for mild, *n* = 12 for moderate, *n* = 5 for severe grade).

### Univariate comparison of outcome variables and GvHD incidence after allo-HCT

**Fig. 2:**
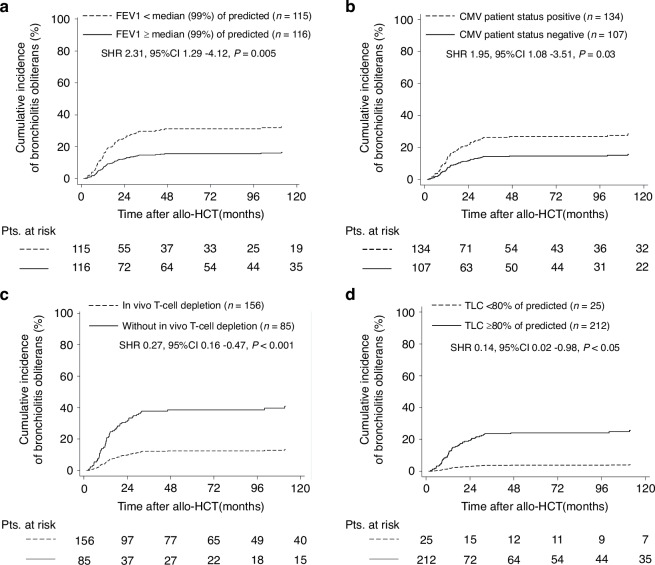
Cumulative incidence of BOS by clinical factors and lung function tests associated with BOS in multivariate analysis. Cumulative incidence curve represent bronchiolitis obliterans by (**a**) FEV1 ≥ or <median (99% of predicted), (**b**) CMV patient positivity or negativity, (**c**) GvHD prophylaxis with or without in vivo T-cell depletion and (**d**) TLC ≥ or < 80% of predicted in patients conditioned with myeloablative conditioning regimens prior allo-HCT. Statistical analysis was performed for Fine and Gray regression models in the presence of competing risks. FEV1 forced expiratory volume in 1 s., TLC total lung capacity, Pts patients, allo-HCT allogeneic hematopoietic cell transplantation, SHR subdistribution hazard ratio, CI confidence intervals.

**Fig. 3:**
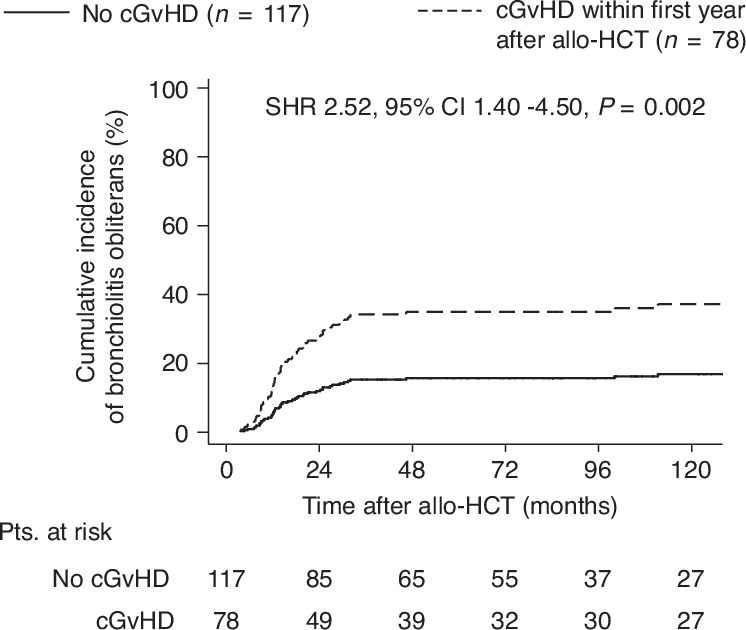
Cumulative incidence of BOS by cGvHD within the first year after allo-HCT. We performed a landmark analysis of cGvHD excluding BOS as risk factor for BOS development. Patients surviving 365 days after allo-HCT were included in the analysis (*n* = 195). Cumulative incidence of BOS was analyzed by cGvHD onset within the first year after allo-HCT. SHR subdistribution hazard risk, CI confidence interval, cGvHD chronic GvHD, allo-HCT allogeneic hematopoietic cell transplantation.

We analyzed outcome variables and GvHD incidence by myeloablative conditioning in univariate analysis (Supplementary Figs. 2–3, Supplementary Table 3). Some differences were observed such as a trend for improved OS (HR 0.50, *p* = 0.06), PFS (HR 0.51, p = 0.05), a decreased relapse incidence (Fig. 2, SHR 0.47, *p* = 0.06) and a trend to lower incidence of cGvHD (Fig. 3, HR 0.52, *p* = 0.05) in patients treated with FluBu4 compared to patients conditioned with BuCy. No significant differences were observed in outcome variables between patients treated with TBF MAC and BuCy. These results should be interpreted with caution due to the nature of univariate analysis, lower number of patients transplanted with FluBu4 (*n* = 29) and unbalanced clinical features pre-allo-HCT.

### Univariate and multivariate analysis for risk factors associated with BOS

To identify clinical factors and pulmonary parameters associated with BOS in patients treated with myeloablative conditioning, we performed Fine and Gray regression model in univariate (Supplementary Table 4–5) and multivariate analysis including conditioning regimens as hypothesis confounding variable (Table 2A).

**Table 2:** Cox regression multivariate analysis for lung function parameters before allo-HCT and clinical parameters for bronchiolitis obliterans syndrome and death.

A.				
Subdistribution hazard ratio for BOS incidence				
	*N*	*p* value	SHR	95% CI
Conditioning with BuCy FluBu4 TBF MAC	175 29 37	– 0.08 0.77	1 0.28 1.11	– 0.07, 1.17 0.52, 2.40
TLC < 80% predicted	25	0.02	0.12	0.02, 0.71
FEV1 < median % of predicted (99%)	115	0.004	2.39	1.31, 4.35
In vivo T-cell depletion^a^	110	0.001	0.29	0.16, 0.51
CMV patient positivity	134	0.014	2.11	1.16, 3.84

B.				
Hazard ratio estimates for death				
	n	*p* value	HR	95% CI
Conditioning with BuCy FluBu4 TBF MAC	175 29 37	– 0.17 0.76	1 0.60 0.92	– 0.29, 1.24 0.54, 1.55
FEV1 < 80% of predicted initial	31	0.005	2.01	1.23, 3.28
Disease status before allo-HCT^b^ - early - intermediate - late	113 15 111	– 0.56 0.001	1 0.75 2.53	– 0.29, 1.94 1.74, 3.68

Subdistribution hazard ratios (SHR) and confidence intervals (CI) for single pulmonary function test (PFT) values before allo-HCT and clinical parameters were estimated in multivariate analysis following backward selection for (**A**) BOS incidence using the Fine and Gray regression model in the presence of competing risks and for (**B**) overall survival using Cox regression proportional hazard ratio. Single PFT values and clinical parameters with a *p* value of 0.1 in univariate analysis were selected for the multivariate analysis. Conditioning was included as hypothesis variable. FEV1, forced vital capacity in 1 s. BuCy, Busulfan/Cyclophosphamide; FluBu4, Fludarabine/Busulfan 4 days; TBF MAC, Thiotepa/Busulfan/Fludarabine myeloablative conditioning.

^a^In vivo T-cell depletion includes patients receiving alemtuzumab and antithymocyte globuline (ATG).

^b^Disease stage defined by EBMT score (Gratwohl et al., 2012).

In multivariate analysis, we identified FEV1<median (99% of predicted) (HR 2.39, *p* = 0.004) and CMV patient positivity (HR 2.11, *p* = 0.014) to be associated with BOS incidence. In vivo T-cell depletion with alemtuzumab and ATG was shown to be protective (SHR 0.29, *p* = 0.001). Unexpectedly, patients with restrictive lung disease defined as TLC < 80% showed lower incidence of BOS (SHR 0.12, *p* = 0.02) (Table 2A, Fig. 2). BOS incidence did not differ among the three conditioning groups in multivariate analysis (for FluBu4, SHR 0.77, *p* = 0.08; for TBF MAC, SHR 1.11, *p* = 0.28 compared to BuCy) (Table 2A, Supplementary Fig. 1).

Next, we focused on specific components of PFT for early diagnosis of BOS and their predictive capacity. Therefore, we performed a landmark analysis of patients surviving at least 100 days and with available PFTs within the first 100 days after allo-HCT. Several lung function parameters at day +100 after allo-HCT such as FEV1/FVC < 0.7 ratio, RV/TLC > 0.45 ratio, FEV1<median (99% of predicted) and changes in small airways as MEF50 < 50% predicted, MEF50 < 35% predicted, MEF25 < 35% predicted and MEF25 < 25% predicted were also associated with time to BOS in univariate analysis (Table 3). The median time from identified PFT parameters to BOS diagnosis ranged between 164 days (FEV1< median 99% of predicated) and 393 days (MEF25 < 35% of predicted).

**Table 3:** Subdistribution hazard ratios for single lung function parameters after allo-HCT at day 100 for BOS incidence.

Subsdistribution hazard ratio estimates for BOS incidence of patients surviving at least 100 days with available PFTs (*n* = 200/245)										Median time from selected PFT parameters d + 100 to BOS (days)
		Parameter before allo-HCT				Parameter at day 100 after allo-HCT				
		*n*	*p* value	SHR	95% CI	*n*	*p* value	SHR	95% CI	
FEV1/FVC < 0.80 ratio		72	0.35	1.31	0.75, 2.30	86	0.08	1.63	0.94, 2.82	268
FEV1/FVC < 0.70 ratio		12	0.11	2.17	0.84, 5.73	18	0.001	3.47	1.68, 7.14	291
FEV1 < median (99% of predicted)		90	0.002	2.54	1.41, 4.58	115	0.001	4.10	2.00, 8.43	164
FEV1 < 75% predicted		8	0.12	2.20	0.82, 5.84	23	0.03	2.25	1.09, 4.66	386
RV > 120% predicted		66	0.37	1.29	0.74, 2.25	63	0.97	0.98	0.54, 1.78	171
RV/TLC > 0.45 ratio		13	0.86	0.90	0.27, 2.97	22	0.02	2.57	1.23, 5.36	182
TLC < 80% predicted		15	0.11	0.21	0.31, 1.41	34	0.14	1.64	0.84, 3.18	329
MEF50 < 50% predicted		30	0.12	1.66	0.87, 3.17	43	0.001	3.17	1.82, 5.51	379
MEF50 < 35% predicted		6	0.67	1.30	0.38, 4.50	14	0.001	5.19	2.58, 10.4	350
MEF25 < 35% predicted		11	0.41	1.48	0.57, 3.84	62	0.001	3.29	1.89, 5.72	393
MEF25 < 25% predicted		6	0.67	1.30	0.38, 4.50	24	0.001	3.42	1.88, 6.23	277
DLCOc SB < 80% predicted		97	0.44	1.28	0.68, 2.39	97	0.12	1.58	0.88, 2.83	271
DLCOc SB < 60% predicted		20	0.50	1.35	0.55, 3.32	20	0.52	1.22	0.66, 2.24	321

Patients surviving 100 days and with available pulmonary function tests were selected for the analysis (200/245, 82%). Subdistribution hazard ratios (SHR) and confidence intervals (CI) for single pulmonary function test (PFT) values at day 100 after allo-HCT were estimated for BOS in univariate analysis using the Fine and Gray regression model in the presence of competing risks. Cut-off values for PFT were chosen from the significant PFT parameters before allo-HCT and previous publications (Duque-Afonso et al. 2018). Only patients surviving 100 days after allo-HCT and with pulmonary function test (PFT) within the first 100 days after allo-HCT were included in this analysis. Median time from selected PFT parameters from d + 100 to BOS was calculated only for patients developing BOS.

*BOS* bronchiolitis obliterans syndrome, *PFT* pulmonary function tests, *allo-HCT* allogenic hematopoietic cell transplantation, *FEV1* forced expiratory volume in 1 second (FEV1), *FVC* forced vital capacity, *RV* residual volume, *TLC* total lung capacity, *MEF50* mid-expiratory flow 50%, *MEF25* mid-expiratory flow 25%, *DLCOc SB* carbon monoxide diffusion capacity corrected for hemoglobin.

To address whether cGvHD excluding BOS is associated with BOS development in our cohort, we performed a landmark analysis for patients surviving at least one year and we compared the cumulative incidence of BOS by the presence of cGvHD within the first year after allo-HCT. As expected, we identified cGvHD excluding BOS as a risk factor for the development of BOS (SHR = 2.51, 95%CI 1.40–4.50, *p* = 0.002) (Fig. 3).

### Univariate and multivariate analysis for risk factors associated with death

Regarding cause-specific hazard ratios for death, several clinical and PFT parameters were found to be associated with death in univariate analysis prior allo-HCT and at day +100 after allo-HCT (Supplementary Tables 6–8). In multivariate analysis, FEV1 < 80% of predicted (HR 2.01, *p* = 0.005) and advanced disease status before allo-HCT (HR 2.53, *p* = 0.001) were associated with increased risk of death (Table 2B).

The causes for death are described in Table 4: 58% patients have died during the follow up in the BuCy cohort, 27% in the FluBu4 and 48% in the TBF MAC cohort. The predominant reason for death was relapse or progression of the original disease (45%, 21%, 35%, in BuCy, FluBu4 and TBF MAC, respectively). A pulmonary cause of death was observed in 6% (*n* = 12) patients in the BuCy cohort, 10 of these patients died because of infectious pulmonary diseases, 1 because of ARDS and 1 because of bronchiolitis obliterans. In the TBF MAC group, 8% (*n* = 3 patients) died because of infectious pulmonary diseases, whereas in the FluBu4 cohort no deaths due to pulmonary diseases were reported.

**Table 4:** Cause of death.

	BuCy	FluBu4	TBF MAC
***N***	**175**	**29**	**37**
Alive, n (%)	74 (42)	21 (72)	19 (51)
Total deaths, n (%)	101 (58)	8 (27)	18 (48)
Relapse or progression of original disease, n (%)	78 (45)	6 (21)	13 (35)
NRM, pulmonary cause of death, n (%)	12 (6) - bacterial Pneumonia (3x) - Aspergillus pneumonia (3x) - Viral pneumonia (COVID, parainfluenza, influenza, CMV) (4x) - bronchiolitis obliterans - ARDS	0	3 (8) - bact. Pneumonia - Viral pneumonia - CMV pneumonia
NRM, non-pulmonary cause of death, n (%)	9 (5) - secondary malignancy (4x) - cardiac infarction - prior malignancy (breast cancer) - cerebral infarction - cGvHD - Sepsis P. aeroginosa	2 (6) - cGvHD - aGvHD	2 (5) - cGvHD - Secondary malignancy
Unknown, n (%)	2 (2)	0	0

*N* total patient number, *allo-HCT* allogeneic hematopoietic cell transplantation, *BuCy* Busulfan/Cyclophosphamide, *FluBu4* Fludarabine/Busulfan 4 days, *TBF MAC* Thiotepa/Busulfan/Fludarabine myeloablative conditioning.

## Discussion

BOS development after allo-HCT is still an unsolved clinical problem because once established it is very difficult to treat [3, 4]. In addition, the development of risk stratification tools for BOS risk is challenging. In our previous studies, we have focused on the impact of pulmonary function prior allo-HCT on clinical outcomes such as mortality and respiratory failure. In these studies, we examined mainly older patients or with comorbidities conditioned with reduced toxicity/intensity conditioning protocols as FBM and FTM [25, 26]. Our results suggested that patients with moderate small airway disease prior to allo-HCT as depicted by MEF25 < 35% of predicted and MEF50 < 50% of predicted have a higher risk of BOS. In addition, severe small airway disease, decreased CO-diffusion capacity prior allo-HCT as well as a combined restrictive/obstructive lung disease at day +100 after allo-HCT were associated with higher risk for NRM [25].

In the present study, we included a younger patient population receiving a myeloablative conditioning. Therefore, these patients were potentially exposed to less toxic substances (chemotherapy, smoking) and thus rather preserved lung function as compared to patients receiving reduced toxicity conditioning, which are older and/or have higher a comorbidity index. As expected, clinical risk factors and PFT parameters associated with BOS and death were different between both cohorts (Table 1 and Supplementary Table 1).

These striking differences may be partly explained by the different characteristics of the treatment cohorts: First, due to institutional practice at our center, the BuCy conditioning was used up to 2012 and included 175 patients, whereas the more contemporary conditioning FluBu4 included 29 patients and the TBF MAC included 37 patients. We observed the highest prevalence of pre-existent lung disease and smoking in patients treated with BuCy. We found that the MEF25% of predicted and arterial O_2_ in the BuCy cohort was significant lower compared to the other conditioning cohorts. Other variables, which might have contributed to BOS incidence, are lung toxicity by the conditioning therapy itself and different GvHD prophylaxis strategies.

Regarding in vivo TCD, we found that ATG was used more frequently in patients receiving FluBu4 and TBF MAC compared to the BuCy group, in which alemtuzumab was more frequently used. In a multivariate analysis, we observed that in vivo TCD was linked to decreased incidence of BOS. Along with this, we also observed in univariate analysis that patients with an unrelated donor or with an HLA non-identical donor had also a decreased risk of developing BOS. ATG or alemtuzumab are frequently used in this setting to prevent GVHD: 149/161 (92.5%) patients with unrelated donor (*p* < 0.001) and 50/56 (89.2%) patients with HLA-non identical donor received ATG or alemtuzumab (*p* < 0.001) in our cohort. In multivariate analysis, we found a decreased risk for BOS in patients receiving in vivo T-cell depletion with ATG or alemtuzumab but no association was found for patients receiving a graft from unrelated donor or from HLA non-identical donor. These findings are in line with retrospective studies showing that ATG decreased the risk for BOS and a prospective study observing, that in vivo TCD with ATG also protected from chronic lung dysfunction [36, 37].

Moreover, through multivariate analysis, we also identified CMV patient positivity prior allo-HCT and FEV1<median (99)% of predicted prior allo-HCT as a risk factor for the development of BOS. These findings are in line with a retrospective analysis also demonstrating an association between CMV positivity and the development of BOS in multivariate analysis [38]. The same study also found an association between a reduced FEV1 at the time of transplant and the diagnosis of BOS. Interestingly, another study suggested an association between CMV pneumonitis and the development of BOS in patients undergoing lung transplantation [39]. However, so far, the association between CMV infection and the development of chronic GvHD remains controversial [40–42]. The routine use of letermovir prophylaxis for CMV positive patients questions additionally whether CMV positivity is still a risk for the development of BOS.

In multivariate analysis we also found TLC < 80% of predicted to be protective against the development of BOS. This seems to be counterintuitive at first. We hypothesize that, restriction might mask obstructive changes due to increased stiffness of lung parenchyma avoiding further remodeling of airways in this patient population [43, 44]. Interestingly, reduced MEF50 and MEF25 were not associated with time to BOS in multivariate analysis. These findings are in contrast to our study of patients receiving intermediate TCI score protocols FBM/FTM [25]. It has been demonstrated the prevalence of small airway disease, indicated by reduced MEF, increases with age [45, 46]. Therefore, our findings suggest that reduced MEF as a risk factor for the development of BOS in older patients.

Some parameters as FEV1 < 80% of predicted and advanced disease status were identified in multivariate analysis to be associated with increased risk of death. The association of FEV1 < 80% of predicted is in line with the HCT-CI score, in which FEV1 < 80% of predicted is a risk factor for dismal outcome following allo-HCT [15].

One of the strengths of our work is the relative long follow-up, which identified patients developing BOS at later time point after the allo-HCT. To address which risk factors are associated with late onset of BOS, we compared clinical characteristics and PFT parameters prior allo-HCT of patients developing BOS 2 years after allo-HCT with those developing BOS within the first 2 years after allo-HCT (Supplementary Table 9). Interestingly, we found male donor (*p* = 0.049) and in vivo GvHD prophylaxis with alemtuzumab (*p* = 0.01) with a decreased risk for late-onset of BOS.

This study has also some limitations: First, our data was analyzed retrospectively and is derived from a single-center experience, therefore sample errors cannot be excluded. Importantly, due to practice changes over time the number of patients in the different conditioning cohorts is very different with relatively small patient numbers in the FluBu4 and TBF MAC cohort. Patients transplanted between 1998 and 2012 received BuCy and from 2014 to 2019 received FluBu4 and TBF MAC. This might cause a time bias as allo-HCT might have improved over time. These practice changes also led to unbalanced clinical characteristics between the conditioning cohorts, which had different follow up periods. Hence, the BuCy cohort has longer follow up than the TBF MAC and FluBu4 cohort, which limit the conclusions for the comparison of the protocols. Lastly, we focus our work on patients with myeloid malignancies receiving MAC chemotherapy. We excluded patients with lymphoid malignancies receiving total body irradiation as part of the MAC conditioning. Future studies should address the identification of clinical and PFT risk factors in this patient cohort and should compare risk factors from chemotherapy-based with TBI-based MAC conditioning.

In conclusion, we identify and describe clinical factors and PFT parameters prior and after allo-HCT that seem to influence the incidence of BOS after allo-HCT. The identification of risk factors associated with BOS and death might serve to establish pre-emptive and early therapeutic interventions.

## Supplementary information

Supplementary Material

## Data Availability

The datasets generated during and/or analyzed during the current study are available upon reasonable request from the corresponding authors. This article contains supplementary material.
