# Supplementary material for: Evaluation of risk for bronchiolitis obliterans syndrome after allogeneic hematopoietic cell transplantation with myeloablative conditioning regimens
Source: Bone Marrow Transplant. 2024 Sep 27;59(12):1744–53. doi: 10.1038/s41409-024-02422-z (PMC11611741; doi:10.1038/s41409-024-02422-z)
Supplement: Supplementary file 1 — Supplementary Material [file 41409_2024_2422_MOESM1_ESM.docx]

**Supplementary Figures**

**Supplementary Figure 1. Cumulative incidence of bronchiolitis obliterans by myeloablative conditioning regimen prior allo-HCT.** Cumulative incidence curve represents bronchiolitis obliterans in patients conditioned with myeloablative conditioning regimens prior allo-HCT. BuCy, Busulfan/Cyclophosphamide; FluBu4, Fludarabine/Busulfan 4 days: TBF MAC, Thiotepa/Busulfan/Fludarabine myeloablative conditioning, Pts, patients, allo-HCT, allogeneic hematopoietic cell transplantation.

**Supplementary Figure 2.** **Outcome variables by myeloablative conditioning regimen prior allo-HCT**. Kaplan-Meier curves represent **(A)** overall survival and **(B)** progression-free survival and cumulative incidence curve represent **(C)** non-relapse mortality and **(D)** relapse incidence in patients conditioned with myeloablative conditioning regimens prior allo-HCT. BuCy, Busulfan/Cyclophosphamide; FluBu4, Fludarabine/Busulfan 4 days; TBF MAC, Thiotepa/Busulfan/Fludarabine myeloablative conditioning, PTs, patients, allo-HCT, allogeneic hematopoietic cell transplantation.

**Supplementary Figure 3. Cumulative incidence of acute and chronic GvHD by myeloablative conditioning regimen prior allo-HCT.** Cumulative incidence curve represent **(A)** acute and **(B)** chronic GvHD incidence in patients conditioned with myeloablative conditioning regimens prior allo-HCT. BuCy, Busulfan/Cyclophosphamide; FluBu4, Fludarabine/Busulfan 4 days; TBF MAC, Thiotepa/Busulfan/Fludarabine myeloablative conditioning, PTs, patients, allo-HCT, allogeneic hematopoietic cell transplantation, aGvHD, acute graft-versus-host disease; cGvHD, chronic graft-versus-host disease.

|  | **BuCy** | **FluBu4** | **TBF MAC** | **p-value** |
| --- | --- | --- | --- | --- |
| N | 175 | 29 | 37 |  |
| Underlying hematological disease, n (%)   - AML - MDS/MPN | 163 (93.1)  12 (6.9) | 29 (100)  0 | 37 (100)  0 | 0.11 |
| Therapy linked disorder, n (%) | 10 (5.7) | 0 | 1 (3) | 0.33 |
| HCT-CI score, n(%)   - 0 - 1-2 - 3-4 - ≥5 | 154 (88)  10 (6)  7 (4)  4 (2) | 15 (52)  11 (38)  3 (2)  0 | 14 (38)  12 (32)  9 (24)  2 (5) | 0.001 |
| EBMT disease risk score, n (%)   - 0-2 - 3-4 - 5-7 | 40 (23)  80 (46)  55 (31) | 21 (72)  8 (28)  0 | 18 (49)  19 (51)  0 | 0.001 |
| Disease stage at transplant^1^, n (%)   - early - intermediate - late | 79 (45)  12 (7)  84 (48) | 19 (66)  2 (7)  8 (27) | 15 (40)  1 (3)  19 (51) | 0.21 |
| CMV donor positivity, n (%) | 79 (45) | 14 (48) | 13 (35) | 0.48 |
| CMV patient positivity, n (%) | 103 (98) | 13 (45) | 18 (49) | 0.24 |
| CMV reactivation, n (%) | 67 (38) | 9 (31) | 15 (40) | 0.70 |
| aGvHD, organ involved, n (%)   - skin - gut - liver | 77 (44)  4 (2)  7 (4) | 10 (35)  6 (21)  3 (10) | 22 (59)  10 (27)  5 (14) | 0.11  0.001  0.05 |
| aGvHD grade   - 0 - 1 - 2 - ≥3 | 88 (50)  36 (21)  31 (17)  20 (11) | 14 (48)  5 (17)  4 (3)  6 (7) | 13 (35)  9 (24)  4 (11)  11 (30) | 0.13 |
| cGvHD, organ involved, n (%)   - skin - gut - liver - eye - musculo-skeletal - wasting syndrome | 62 (35)  44 (25)  46 (26)  20 (11)  12 (7)  12 (7) | 5 (17)  2 (7)  8 (27)  0  7 (21)  0 | 10 (27)  1 (3)  8 (22)  1 (3)  12 (32)  0 | 0.12  0.001  0.82  0.008  0.001  0.02 |
| cGvHD grade, n (%)   - no cGvHD - mild - moderate - severe | 91 (52)  29 (17)  32 (18)  23 (13) | 17 (59)  2 (7)  10 (35)  0 | 25 (68)  1 (3)  11 (30)  0 | 0.003 |

**Supplementary Table 1. Clinical characteristics at the time of and after allo-HCT.** Abbreviations: AML, acute myeloid leukemia; MDS, myelodysplastic syndrome; MPS, myeloproliferative syndrome; ALL, acute lymphoblastic leukemia, CLL, chronic lymphocytic leukemia; NHL, non-hodgkin lymphoma; MM, multiple myeloma; CML, chronic myeloid leukemia; CyA, cyclosporine A; ATG, anti-thymocyte globuline; HCT-CI, hematopoietic cell transplantation co-morbidity index; EBMT, European Bone Marrow Transplantation; aGvHD, acute Graft-versus-Host-Disease; cGvHD, chronic Graft-versus-Host-Disease. ^1^ Disease stage defined by EBMT disease risk score.

|  | **Only patients with BOS (n=54)** | | |
| --- | --- | --- | --- |
|  | **Before allo-HCT** | **BOS diagnosis** | **p-value** |
| RV (% of predicted),  median (range) | 114  (82 - 170) | 169  (93 - 298) | 0.001 |
| RV/TLC ratio  median (range) | 0.34  (0.20 - 1.18) | 0.48  (0.37 - 2.15) | 0.001 |
| VCmax (% of predicted)  median (range) | 96  (69 - 134) | 73  (24 - 104) | 0.001 |
| TLC (% of predicted)  median (range) | 97  (75 -133) | 92  (41 - 135) | 0.13 |
| FEV1 (% of predicted),  median (range) | 95  (70 - 142) | 47  (19 - 81) | 0.001 |
| FEV1/VCmax ratio  median (range) | 0.78  (0.53 - 1.16) | 0.61  (0.40 - 1.44) | 0.001 |
| FEV1/FVC ratio,  median (range) | 0.82  (0.64 - 1.00) | 0.61  (0.40 - 0.92) | 0.001 |
| FVC (% of predicted),  median (range) | 96  (59 - 138) | 69  (17 - 105) | 0.001 |
| MEF50 (% of predicted),  median (range) | 68  (4 - 136) | 17  (1 - 71) | 0.001 |
| MEF25 (% of predicted),  median (range) | 41  (11 - 103) | 11  (0 - 88) | 0.001 |
| aCO2 (mmHg), absolute,  median (range) | 38  (30 - 44) | 36  (24 - 45) | 0.01 |
| aO2 (mmHg), absolute,  median (range) | 81  (65 - 102) | 70  (51 - 95) | 0.001 |
| DLCOc SB (% of predicted), median (range) | 74  (52 - 99) | 57  (36 - 94) | 0.001 |

**Supplementary Table 2. Pulmonary function tests before allo-HCT and at BOS diagnosis in patients after allo-HCT.** Abbreviations: Allo-HCT, allogeneic hematopoietic cell transplantation; BOS, bronchiolitis obliterans syndrome; PFTs, pulmonary function tests; RV, residual volume; TLC, total lung capacity; VC, vital capacity; FEV1, forced expiratory volume in 1 second (FEV1); FVC, forced vital capacity; MEF50, mid-expiratory flow 50%; MEF25, mid-expiratory flow 25%; aCO2, arterial CO2; aO2, arterial O2; DLCOc SB, carbon monoxide diffusion capacity corrected for hemoglobin. Statistical analysis was performed by Wilcoxon matched-pairs signed-rank test.

| **Outcome variable** | **Conditioning** | **p value** | **HR/SHR** | **95% CI** |
| --- | --- | --- | --- | --- |
| Overall survival | BuCy  FluBu4  TBF MAC | 0.06  0.87 | 1  0.50  1.04 | -  0.24 - 1.02  0.63 - 1.71 |
| Progression-free survival | BuCy  FluBu4  TBF MAC | 0.05  0.97 | 1  0.51  0.99 | -  0.26 - 1.00  0.61 - 1.60 |
| Relapse incidence | BuCy  FluBu4  TBF MAC | 0.06  0.31 | 1  0.47  0.74 | -  0.22 - 1.01  0.43 - 1.30 |
| Non-relapse mortality | BuCy  FluBu4  TBF MAC | 0.68  0.47 | 1  0.74  1.42 | -  0.17 - 3.15  0.54 - 3.73 |
| aGvHD | BuCy  FluBu4  TBF MAC | 0.16  0.75 | 1  0.66  1.08 | -  0.38 - 1.17  0.66 - 1.78 |
| BOS | BuCy  FluBu4  TBF MAC | 0.07  0.51 | 1  0.26  0.76 | -  0.06 - 1.12  0.34 - 1.70 |
| cGvHD | BuCy  FluBu4  TBF MAC | 0.05  0.22 | 1  0.52  0.72 | -  0.27 - 1.00  0.42 - 1.21 |

**Supplementary Table 3.** **Univariate analysis for outcome variables by myeloablative conditioning.** Hazard Hazard ratios (HR) and confidence intervals were estimated for overall survival and progression-free survival in univariate analysis using Cox proportional hazards regression model. Subdistribution hazard ratios (SHR) and CI were estimated for relapse, non-relapse mortality, aGvHD, BOS, cGvHD in univariate analysis using the Fine and Gray regression model in the presence of competing risks. n, number of patients fulfilling the PFT parameter. aGvHD, acute graft-versus-host-disease; BOS, bronchiolitis obliterans; cGvHD, chronic graft-versus-host-disease. BuCy, Busulfan/Cyclophosphamide; FluBu4, Fludarabine/Busulfan 4 days; TBF MAC, Thiotepa/Busulfan/Fludarabine myeloablative conditioning.

| **Subdistribution hazard ratio estimates for BOS incidence** | | | | |
| --- | --- | --- | --- | --- |
| **Clinical parameters** | **n** | **p value** | **SHR** | **95% CI** |
| Patient sex female | 107 | 0.29 | 0.74 | 0.43, 1.30 |
| Donor sex female | 102 | 0.42 | 1.25 | 0.73, 2.14 |
| Antecedent hematological disorder | 41 | 0.02 | 2.07 | 1.14, 3.76 |
| Therapy linked disorder | 11 | 0.06 | 2.17 | 0.97, 4.87 |
| Diseases remission status   - controlled - uncontrolled - untreated | 127  99  14 | 1  0.53  0.34 | -  0.83  1.56 | -  0.46, 1.48  0.62, 3.91 |
| Donor unrelated | 161 | 0.001 | 0.33 | 0.19, 0.57 |
| Donor HLA non-identical | 56 | 0.02 | 0.33 | 0.13, 0.85 |
| HLA-C different | 34 | 0.78 | 1.11 | 0.52, 2.36 |
| GvHD prophylaxis with MTX | 127 | 0.004 | 2.39 | 1.31, 4.35 |
| GvHD prophylaxis with in vivo T cell depletion^1^ | 156 | 0.001 | 0.27 | 0.15, 0.47 |
| Smoking | 58 | 0.23 | 1.43 | 0.79, 2.58 |
| Lung disease before allo-HCT^2^ | 82 | 0.49 | 0.81 | 0.46, 1.44 |
| Lung disease after allo-HCT^3^ | 61 | 0.09 | 1.64 | 0.92, 2.91 |
| HCT-CI score ≥ 1 | 42 | 0.93 | 0.97 | 0.51, 1.86 |
| HCT-CI score ≥ 2 | 25 | 0.75 | 0.88 | 0.41, 1.90 |
| HCT-CI score ≥ 3 | 10 | 0.81 | 0.90 | 0.36, 2.23 |
| HCT-CI score ≥ 4 | 5 | 1.48 | 1.49 | 0.48, 4.58 |
| EBMT disease risk score   - 0-2 - 3-4 - 5-7 | 79  107  55 | -  0.83  0.99 | 1  1.07  0.99 | -  0.57, 2.00  0.48, 2.04 |
| Disease status before allo-HCT   - early - intermediate - late | 113  15  111 | -  0.46  0.39 | 1  1.42  0.78 | -  0.56, 3.59  0.44, 1.37 |
| Karnofsky ≤ 90 | 190 | 0.30 | 1.43 | 0.72, 2.88 |
| Karnofsky ≤ 80 | 53 | 0.49 | 0.79 | 0.39, 1.56 |
| Karnofsky ≤ 70 | 18 | 0.95 | 1.03 | 0.36, 2.95 |
| CMV donor positivity | 106 | 0.39 | 1.26 | 0.73, 2.16 |
| CMV patient positivity | 134 | 0.03 | 1.95 | 1.08, 3.52 |
| CMV reactivation | 91 | 0.89 | 0.96 | 0.55, 1.67 |

**Supplementary Table 4. Subdistribution hazard ratios for bronchiolitis obliterans syndrome (BOS) of clinical parameters before allo-HCT.** Subdistribution hazard ratios (SHR) and confidence intervals (CI) for clinical parameters were estimated for BOS incidence in univariate analysis using the Fine and Gray regression model in the presence of competing risks. n, number of patients fulfilling the PFT parameter. ^1^ Patients, who did receive cyclosporine A/anti-thymocyte globulin or cyclosporine A/alemtuzumab before allo-HCT were included in this group.^2^ Lung diseases before allo-HCT and ^3^ lung diseases after allo-HCT are described in Table 1.

| **Subdistribution hazard ratio estimates for BOS incidence** | | | | |
| --- | --- | --- | --- | --- |
| **PFT values** | **n** | **p value** | **SHR** | **95% CI** |
| RV >120% predicted | 82 | 0.68 | 1.12 | 0.64, 1.94 |
| TLC < 80% predicted | 25 | 0.05 | 0.14 | 0.02, 0.98 |
| RV/TLC ratio > 40% ratio | 36 | 0.69 | 0.84 | 0.38, 1.88 |
| FEV1 < median (99%) | 115 | 0.005 | 2.31 | 1.29, 4.13 |
| FEV1 < 80% of predicted | 31 | 0.17 | 1.59 | 0.81, 3.13 |
| FEV1/VCmax < 70% ratio | 20 | 0.007 | 2.58 | 1.28, 5.15 |
| FEV1/VCmax < 80% ratio | 102 | 0.09 | 1.61 | 0.94, 2.76 |
| FEV1/FVC < 70% ratio | 17 | 0.12 | 1.99 | 0.83, 4.75 |
| FEV1/FVC < 80% ratio | 83 | 0.11 | 1.56 | 0.91, 2.70 |
| FVC < median (101.5%) | 117 | 0.21 | 1.42 | 0.82, 2.46 |
| FVC < 95 % of predicted | 87 | 0.16 | 1.47 | 0.86, 2.52 |
| FVC < 80 % of predicted | 28 | 0.24 | 0.55 | 0.20, 1.49 |
| MEF50 < median (75%) | 109 | 0.15 | 1.49 | 0.87, 2.57 |
| MEF50 < 50% of predicted | 35 | 0.06 | 1.82 | 0.97, 3.41 |
| MEF25 < median (47.5%) | 105 | 0.008 | 2.13 | 1.22, 3,72 |
| MEF25 < 40% of predicted | 78 | 0.03 | 1.87 | 1.09, 3.21 |
| CO2<median (37 mmHg) | 125 | 0.15 | 0.66 | 0.38, 1.15 |
| O2 < median (84 mm Hg ) | 128 | 0.13 | 1.56 | 0.87, 2.81 |
| DLCOcSB < 80% of predicted | 118 | 0.58 | 1.18 | 0.65, 2.16 |
| DLCOcSB < 60% of predicted | 26 | 0.83 | 0.90 | 0.35, 2.33 |

**Supplementary Table 5. Subddistribution hazard ratios for bronchiolitis obliterans syndrome (BOS) of single pulmonary function tests (PFT) values before allo-HCT.** Subdistribution hazard ratios (SHR) and confidence intervals (CI) for single pulmonary function test (PFT) values before allo-HCT and were estimated for BOS incidence in univariate analysis using the Fine and Gray regression model in the presence of competing risks. Only significant and selected PFT using the minimum p-value approach are shown. n, number of patients fulfilling the PFT parameter.

| **Hazard ratio estimates for death** | | | | |
| --- | --- | --- | --- | --- |
| **Clinical parameters** | **n** | **p value** | **HR** | **95% CI** |
| Conditioning with BuCy  FluBu4  TBF MAC | 175  29  37 | -  0.06  0.88 | 1  0.50  1.04 | -  0.24, 1.02  0.64, 1.71 |
| Patient sex female | 107 | 0.95 | 0.99 | 0.69, 1.39 |
| Donor sex female | 102 | 0.92 | 1.02 | 0.72, 1.44 |
| Antecedent hematological disorder | 41 | 0.80 | 1.06 | 0.68, 1.63 |
| Therapy linked disorder | 11 | 0.14 | 1.62 | 0.85, 3.09 |
| Diseases remission status   - controlled - uncontrolled - untreated | 127  99  14 | 1  0.001  0.94 | -  2.21  0.97 | -  1.55, 3.16  0.44, 2.13 |
| Donor unrelated | 161 | 0.65 | 1.09 | 0.75, 1.57 |
| Donor HLA-non-ident | 56 | 0.10 | 1.39 | 0.94, 2.05 |
| HLA-C different | 34 | 0.18 | 1.37 | 0.86, 2.16 |
| GvHD Prophylaxis MTX | 127 | 0.98 | 0.99 | 0.70, 1.41 |
| GvHD Prophylaxis with in vivo T cell depletion^1^ | 156 | 0.92 | 1.02 | 0.71, 1.45 |
| Smoking | 58 | 0.75 | 1.06 | 0.72, 1.57 |
| Lung disease before allo-HCT^2^ | 82 | 0.26 | 0.81 | 0.56, 1.17 |
| Lung disease after allo-HCT^3^ | 61 | 0.09 | 1.38 | 0.96, 2.01 |
| HCT-CI score ≥ 1 | 58 | 0.64 | 1.10 | 0.73, 1.65 |
| HCT-CI score ≥ 2 | 42 | 0.86 | 1.04 | 0.66, 1.65 |
| HCT-CI score ≥ 3 | 25 | 0.88 | 0.95 | 0.54, 1.70 |
| HCT-CI score ≥ 4 | 10 | 0.42 | 1.37 | 0.64, 2.93 |
| EBMT disease risk score   - 0-2 - 3-4 - 5-7 | 79  107  55 | -  0.001  0.001 | 1  2.51  2.78 | -  1.59, 3.97  1.67, 4.61 |
| Disease status before allo-HCT   - early - intermediate - late | 113  15  111 | -  0.99  0.001 | 1  0.99  2.42 | -  0.45, 2.22  1.68, 3.48 |
| Karnofsky ≤ 90 | 190 | 0.12 | 1.41 | 0.91, 2.19 |
| Karnofsky ≤ 80 | 53 | 0.02 | 1.61 | 1.09, 2.38 |
| Karnofsky ≤ 70 | 18 | 0.07 | 1.72 | 0.97, 3.07 |
| CMV donor positivity | 106 | 0.84 | 0.97 | 0.68, 1.36 |
| CMV patient positivity | 134 | 0.71 | 0.94 | 0.66, 1.32 |
| CMV reactivation | 91 | 0.77 | 1.05 | 0.74, 1.50 |

**Supplementary Table 6. Hazard ratios for death of clinical factors associated to allo-HCT.** Hazard ratios (HR) and confidence intervals (CI) for clinical parameters were estimated for death in univariate analysis using Cox proportional hazards regression model. n, number of patients fulfilling the single and combined PFT parameter. ^1^ Patients, who did receive cyclosporine A/anti-thymocyte globuline or cyclosporine A/alemtuzumab before allo-HCT were included in this group. ^2^Lung diseases before allo-HCT and ^3^lung diseases after allo-HCT are described in Table 1.

| **Hazard ratio estimates for death** | | | | |
| --- | --- | --- | --- | --- |
| **PFT values** | **n** | **p value** | **HR** | **95% CI** |
| FEV1/FVC < 80% ratio | 83 | 0.51 | 0.88 | 0.61, 1.28 |
| FEV1/FVC < 70% ratio | 17 | 0.82 | 1.08 | 0.55, 2.13 |
| FEV1 <median of predicted (99%) | 115 | 0.50 | 1.12 | 0.80, 1.59 |
| FEV1 < 80% of predicted | 31 | 0.07 | 1.56 | 0.98, 2.50 |
| FEV1 < 75% of predicted | 14 | 0.10 | 1.72 | 0.90, 3.28 |
| RV >120% predicted | 82 | 0.32 | 1.20 | 0.84, 1.71 |
| RV/TLC ratio > 40% ratio | 36 | 0.01 | 1.77 | 1.14, 2.72 |
| TLC < 80% predicted | 25 | 0.49 | 1.21 | 0.70, 2.11 |
| FVC < 80 % of predicted | 28 | 0.09 | 1.55 | 0.93, 2.59 |
| MEF50 < 50% of predicted | 35 | 0.76 | 1.07 | 0.67, 1.72 |
| MEF50 < 30% of predicted | 7 | 0.11 | 1.95 | 0.86, 4.46 |
| MEF25 < 35% of predicted | 15 | 0.37 | 1.34 | 0.70, 2.57 |
| MEF25 < 25% of predicted | 7 | 0.09 | 2.06 | 0.91, 4.70 |
| O2 < median (84 mm Hg) | 128 | 0.74 | 0.94 | 0.65, 1.35 |
| CO2 < median (37 mmHg) | 125 | 0.88 | 1.02 | 0.72, 1.47 |
| DLCOcSB < 80% of predicted | 118 | 0.74 | 0.93 | 0.59, 1.44 |
| DLCOcSB < 60% of predicted | 26 | 0.85 | 1.06 | 0.53, 2.14 |

**Supplementary Table 7. Hazard ratios for death of single pulmonary function tests (PFTs) values before allo-HCT.** Hazard ratios (HR) and confidence intervals (CI) for single pulmonary function test (PFT) values before allo-HCT were estimated for death in univariate analysis using Cox proportional hazards regression model. Single PFT values were selected using the minimum p-value approach. n, number of patients fulfilling the single and combined PFT parameter.

| **Hazard ratio estimates for death**  **of patients surviving at least 100 days with available PFTs (n= 200/245)** | | | | |
| --- | --- | --- | --- | --- |
| **Parameter at day 100**  **after allo-HCT** | **n** | **p value** | **HR** | **95% CI** |
| FEV1/FVC <0.80 ratio | 86 | 0.67 | 1.09 | 0.74, 1.62 |
| FEV1/FVC <0.70 ratio | 18 | 0.71 | 1.13 | 0.57, 2.25 |
| FEV1 <75% predicted | 23 | 0.72 | 1.11 | 0.61, 2.04 |
| RV >120% predicted | 63 | 0.21 | 1.30 | 0.87, 1.96 |
| RV/TLC >0.45 ratio | 22 | 0.26 | 1.40 | 0.78, 2.50 |
| TLC <80% predicted | 34 | 0.79 | 1.07 | 0.64, 1.81 |
| MEF50 <50% predicted | 43 | 0.36 | 1.23 | 0.79, 1.95 |
| MEF50 <35% predicted | 14 | 0.05 | 1.94 | 1.00, 3.73 |
| MEF25 <35% predicted | 62 | 0.43 | 1.18 | 0.78, 1.79 |
| MEF25 <25% predicted | 24 | 0.79 | 1.08 | 0.60, 1.94 |
| DLCOc SB <80% predicted | 97 | 0.04 | 1.58 | 1.03, 2.42 |
| DLCOc SB <60% predicted | 20 | 0.009 | 1.76 | 1.15, 2.69 |

**Supplementary Table 8. Hazard ratios for single lung function parameters after allo-HCT at day 100 for death.** Patients surviving 100 days and with available pulmonary function tests were selected for the analysis (200/245, 82%). **(A)** Hazard ratios (HR) and confidence intervals (CI) for single pulmonary function test (PFT) values before allo-HCT were estimated for death in univariate analysis using Cox proportional hazards regression model.

| **Comparison of clinical characteristics in patients developing BOS** | | | |
| --- | --- | --- | --- |
|  | **within first 2 years after allo-HCT** | **after 2 years after allo-HCT** | **p-value** |
| **N** | 43 | 11 |  |
| **Patient sex (% of male)** | 26 (60) | 8 (72) | 0.45 |
| **Donor sex (% of male)** | 26 (60) | 3 (27) | 0.049 |
| **Age at allo-HCT, median (range)** | 45 (20, 54) | 41 (22, 48) | 0.20 |
| **KPS, median (range)** | 90 (30, 100) | 90 (80, 100) | 0.58 |
| **Median follow up in months (range)** | 43 (5, 264) | 105 (29, 247) | 0.47 |
| **Donors, n (%)**   - related - unrelated | 25 (58)  18 (42) | 5 (45)  6 (55) | 0.45 |
| **GvHD prophylaxis, n (%)**   - without *in vivo* TCD - with *in vivo* TCD   **Type of GvHD prophylaxis**, n (%)   - CyA/ATG - CyA/alemtuzumab | 29 (67)  14 (32)  8 (57)  6 (43) | 5 (45)  6 (55)  6 (100)  0 | 0.18  0.01 |
| **Lung disease before allo-HCT, n (%)** | 14 (32) | 3 (27) | 0.74 |
| **Lung disease up to 100 d after allo-HCT, n (%)** | 16 (37) | 2 (18) | 0.23 |
| **Smoking current or previous (%)** | 15 (35) | 2 (18) | 0.29 |
| **Pulmonary function tests before allo-HCT, median (range)**  - FEV1 (% predicted)  - FEV1/FVC ratio  - MEF50 (% predicted)  - MEF25 (% of predicted)  - DLCOc SB (% predicted)  - RV (% predicted)  - RV/TLC ratio  - TLC (% predicted)  - aCO2 (mmHg)  - aO2 (mmHg) | 95 (70, 142)  0.82 (0.64, 1.00)  69 (5, 136)  41 (11, 103)  72 (52, 99)  112 (82, 170)  0.34 (0.20, 1.20)  98 (81, 132)  38 (30, 44)  82 (65, 102) | 93 (70, 103)  0.80 (0.67, 0.99)  59 (24, 90)  39 (18, 99)  76 (54, 154)  117 (95, 154)  0.33 (0.23, 0.44)  94 (75, 108)  38 (30, 41)  81 (73, 94) | 0.14  0.31  0.11  0.66  0.48  0.93  0.44  0.29  0.44  0.83 |
| **Time from allo-HCT to BOS diagnosis in months, median (range)** | 11.7 (2.4, 23.2) | 30.3 (24.9, 111.3) |  |
| **BOS severity, n (%)**   - Mild (FEV1 60-79%) - Moderate (FEV1 40-59%) - Severe (FEV1 ≤39%) | 11 (26)  22 (51)  10 (23) | 3 (27)  5 (45)  3 (27) | 0.94 |

**Supplementary Table 9. Comparison of clinical characteristics and pulmonary function tests of patients developing BOS within vs. after 2 years after allo-HCT .** Abbreviations: Allo-HCT, allogeneic hematopoietic cell transplantation; KPS, Karnofsky performance score; GvHD, graft-versus-host disease; TCD, T-cell depletion; CyA, cyclosporine A; ATG, antithymocyte globuline; BOS, bronchiolitis obliterans syndrome; FEV1, forced expiratory volume in 1 second (FEV1); FVC, forced vital capacity; RV, residual volume; TLC, total lung capacity; MEF50, mid-expiratory flow 50%; MEF25, mid-expiratory flow 25%; DLCOc SB, carbon monoxide diffusion capacity corrected for haemoglobin.
